# Supplementary material for: Content-rich biological network constructed by mining PubMed abstracts
Source: BMC Bioinformatics. 2004 Oct 8;5:147. doi: 10.1186/1471-2105-5-147 (PMC528731; doi:10.1186/1471-2105-5-147)
Supplement: Additional File 2 — The original results of the above study (non-essential files are deleted to keep the file size under the limit set by BMC bioinformatics). [file 1471-2105-5-147-S2.bz2 › chilibotAdditionalFile2/dip05/508ID9920921E10800/html/NR3C1_NRIP1.html]

 


 **NR3C1** and **NRIP1** 
  
Found 9 abstracts in PubMed, retrieved 05.  
 

 What does Google say? 
 PDF only 
| .edu only 

---

**Interactive relationship** (e.g. stimulation, inhibition, etc)

**Inhibitory relationship**- Receptor Interacting Protein 140  [ **NRIP1** ]  Binds c Jun and Inhibits Estradiol Induced Activator Protein 1 Activity by Reversing Glucocorticoid Receptor  [ **NR3C1** ]  Interacting Protein 1 Effect.  Ref: 12554755 Mol Endocrinol, 2003
- Coexpression of other AF 2 interacting proteins, including the p160 factors, steroid receptor coactivator 1a SRC 1a and glucocorticoid receptor  [ **NR3C1** ]  interacting protein 1 GRIP1, had negligible effects on ER alpha Pit 1 cooperative activation, but partially relieved RIP140  [ **NRIP1** ]  inhibition.  Ref: 10379892 Mol Endocrinol, 1999
**Neutral relationship**

**Non-interactive relationship** (e.g. studied together, co-existance, homology, etc.)

- Regulation of glucocorticoid receptor  [ **NR3C1** ]  activity by 14 3 3 dependent intracellular relocalization of the corepressor RIP140  [ **NRIP1** ] .  Ref: 11266503 Mol Endocrinol, 2001
- Receptor interacting protein 140  [ **NRIP1** ]  RIP140  [ **NRIP1** ]  is a corepressor that negatively regulates the ligand induced activity of several nuclear receptors, including the glucocorticoid receptor  [ **NR3C1** ]  GR.  Ref: 12773562 Mol Cell Biol, 2003
